# Parapharyngeal space pleomorphic adenoma: a case report

**DOI:** 10.11604/pamj.2017.26.234.11832

**Published:** 2017-04-25

**Authors:** Moncef Sellami, Slim Charfi

**Affiliations:** 1 Department of Otorhinolaryngology-Head and Neck Surgery Habib Bourguiba University Hospital, Sfax, Tunisia; 2 Sfax Medical School, University of Sfax, Sfax, Tunisia; 3 Department of Anatomopathology, Habib Bourguiba University Hospital, Sfax, Tunisia

**Keywords:** Pleomorphic adenoma, parapharyngeal space, surgery

## Image in medicine

Parapharyngeal space masses account for 0.5% of all head and neck neoplasms. Pleomorphic adenoma arising from the epithelial rests of the salivary tissue in the parapharyngeal space is rare. Computed tomography (CT) and Magnetic Resonance Imaging (MRI) allows a precise topographical localization of the mass and the diagnosis of its nature in the majority of cases. Treatment of parapharyngeal pleomorphic adenoma is primarily surgical excision. Tumor recurrence occurs in less than 10% of patients. We report a case of a 37-year-old man presented with dysphagia and disturbed sleep during night since last 6 months. The intraoral examination found a bulging submucosal mass underneath the right oropharynx crossing the midline and pushing the uvula to theluminal left side. CT and MRI scan revealed a heterogeneously enhancing tumor in the right parapharyngeal space measuring 4.5×5×6.4 cm and causing luminal narrowing of the oropharynx. The patient was operated through a transcervical approach to gain entry into parapharyngeal space without osteotomy of the mandible. The tumor was completely resected without rupture. The postoperative course was uneventful. Histopathological examination confirmed the diagnosis of a pleomorphic adenoma. At the 3 years follow-up the patient was free from disease.

**Figure 1:**
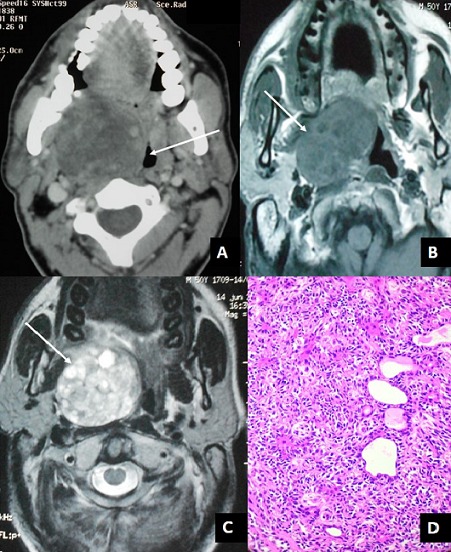
(A) axial computed tomography shows a tumor measuring 4.5×5×6.4 cm in the right parapharyngeal space with significant mass effect (arrow); (B) axial T1-weighted MRI shows a heterogeneous, hypointense tumor in the right parapharyngeal space (arrow); (C) axial T2-weighted MRI shows a heterogeneous, hyperintense tumor (arrow); (D) histopathological examination revealed a mixed epithelial and myoepithelial cells in a myxoid stroma consistent with a pleomorphic adenoma (HE x 400)

